# Complex plasma research under microgravity conditions

**DOI:** 10.1038/s41526-023-00261-8

**Published:** 2023-02-07

**Authors:** Markus. H. Thoma, Hubertus M. Thomas, Christina A. Knapek, Andre Melzer, Uwe Konopka

**Affiliations:** 1 I. Physics Institute, University Giessen, Heinrich-Buff-Ring 16, 35392 Giessen, Germany; 2 DLR Institute of Materials Physics in Space, German Aerospace Center (DLR), Linder Höhe, 51147 Köln, Germany; 3 Institute of Physics, University Greifswald, Felix-Hausdorff-Straße 6, 17489 Greifswald, Germany; 4 Auburn University, 380 Duncan Drive, Auburn, AL 36849 USA

**Keywords:** Plasma physics, Thermodynamics

## Abstract

The future of complex plasma research under microgravity condition, in particular on the International Space Station ISS, is discussed. First, the importance of this research and the benefit of microgravity investigations are summarized. Next, the key knowledge gaps, which could be topics of future microgravity research are identified. Here not only fundamental aspects are proposed but also important applications for lunar exploration as well as artificial intelligence technology are discussed. Finally, short, middle and long-term recommendations for complex plasma research under microgravity are given.

## Introduction

**Fig. 1:**
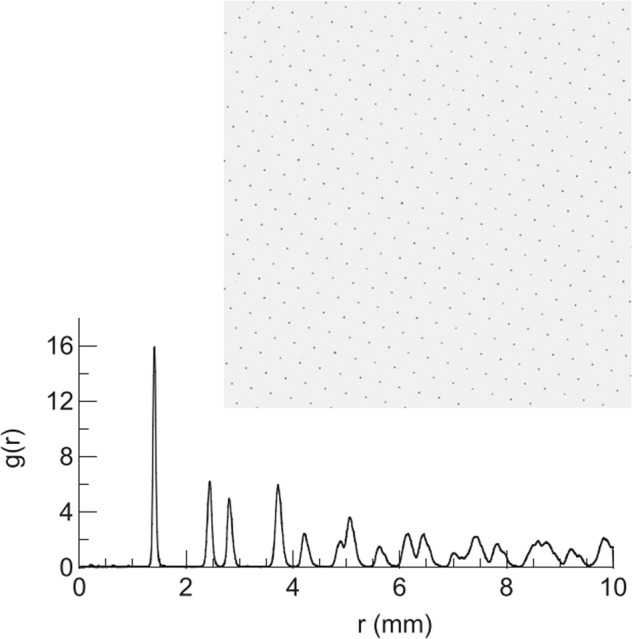
The plasma crystal. Snapshot of a 2D complex plasma crystal showing about 400 particles (upper panel) and the pair correlation function g(r) as a function of the inter-particle distance r (lower panel). The image is inverted and its brightness is adjusted for better viewing^6^.

Complex plasma is a state of soft matter where microparticles are immersed in a weakly ionized gas. The particles acquire a charge in the plasma that scales with the surface potential and the dust size and ranges to 10^3^–10^4^ elementary charges for micrometer-sized particles. This provides a strong Coulomb interaction and therefore strong coupling between the microparticles and allows studying gaseous, liquid and crystalline states of the particle arrangements as well as transitions between them on the individual particle—the kinetic – level^1–5^. For example, a plasma crystal and the corresponding pair correlation function are shown in Fig. 1^6^.

The importance of complex plasma research is based on several aspects:

1. Physical processes in complex plasmas can be studied at the kinetic level: the behavior of individual microparticles can be observed in real time using rather simple optical means. This makes complex plasmas an ideal model system for the investigation of statistical processes in many-particle systems. Moreover, an analysis of the three-dimensional dynamics is accessible.
2. The particle-plasma and the particle-particle interaction can be tuned, controlled and manipulated in various ways (e.g., by changing plasma parameters, applying external electric or magnetic fields, radiation fields, optical tweezers and many more).
  # Corresponding author (markus.h.thoma@physik.jlug.de)
3. Complex plasma offers the opportunity to extend the regime of soft matter research to a virtually undamped system—complementary to the strongly damped colloidal systems. Due to the low charge-to-mass ratio and neutral gas density, characteristic relaxation times in the microparticle component are considerably stretched with respect to normal condensed matter, but still far shorter than in colloids, yielding reasonable observation times.
4. Due to the widespread occurrence of dusty plasma media in nature, increasing the knowledge about it is itself of great interest. Dust and dusty plasmas are ubiquitous in the Universe. They can be found in planetary rings, cometary tails, interplanetary and interstellar clouds, Earth mesosphere, thunderclouds, in the vicinity of spacecrafts and space stations, on planetary surfaces, etc. Extending complex plasma experiments to lunar-like dust will yield results of high importance to future space missions.
5. The presence of dust plays an important role in many technological processes (such as plasma deposition, microelectronic production, etching, where dust is formed during the production process), as well as in thermonuclear fusion (where formation of radioactive and toxic dust is critical for the design of the facilities). These applications can profit considerably from the fundamental knowledge gained in complex plasma experiments.

These unique features make complex plasmas a strongly interdisciplinary research field comprising, among others, condensed matter physics, many body physics or astrophysics^1–5^.

**Fig. 2:**
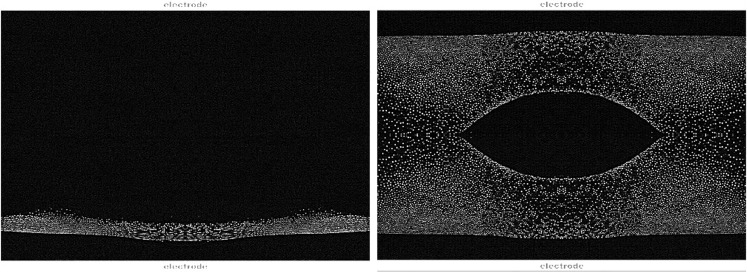
Complex plasma under gravity (left) and microgravity (right) conditions^82^. The microparticle cloud is compressed by gravity (left), whereas the microparticles under microgravity (right) are distributed in the entire plasma chamber apart from a central void.

Gravity strongly affects the behavior of complex plasmas due to the high mass of the microparticles. It forces the microparticles into 2-D, quasi-2-D and stressed 3-D systems. Already this allows fundamental studies of complex plasmas and the list of results is long concerning basic properties (particles charging, pair interaction, waves, etc.), kinetic studies of liquids and solids (liquid-solid phase transitions in 2D and stressed 3D, 2D crystals and crystallization dynamics, defect propagation, etc.), driven systems (hydrodynamic instabilities, shear flow and heat transport in 2D systems, etc.) and anisotropic interactions (active and anisotropic particles)^1–30^. However, to reveal the underlying interactions, homogeneous and isotropic 3D arrangements of the microparticles in the bulk plasma are required. This makes experiments in microgravity mandatory to explore this very special state of matter in its entirety. In Fig. 2 complex plasmas in the stressed state under gravity are compared with homogeneous, extended clouds under microgravity.

**Fig. 3:**
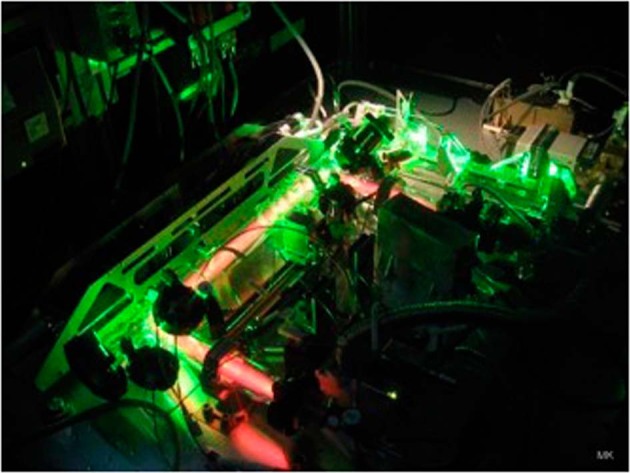
The plasma chamber of the PK-4 experiment. The orange plasma glow in the glass tube of the plasma chamber and the green reflections of the illumination laser are visible.

**Fig. 4:**
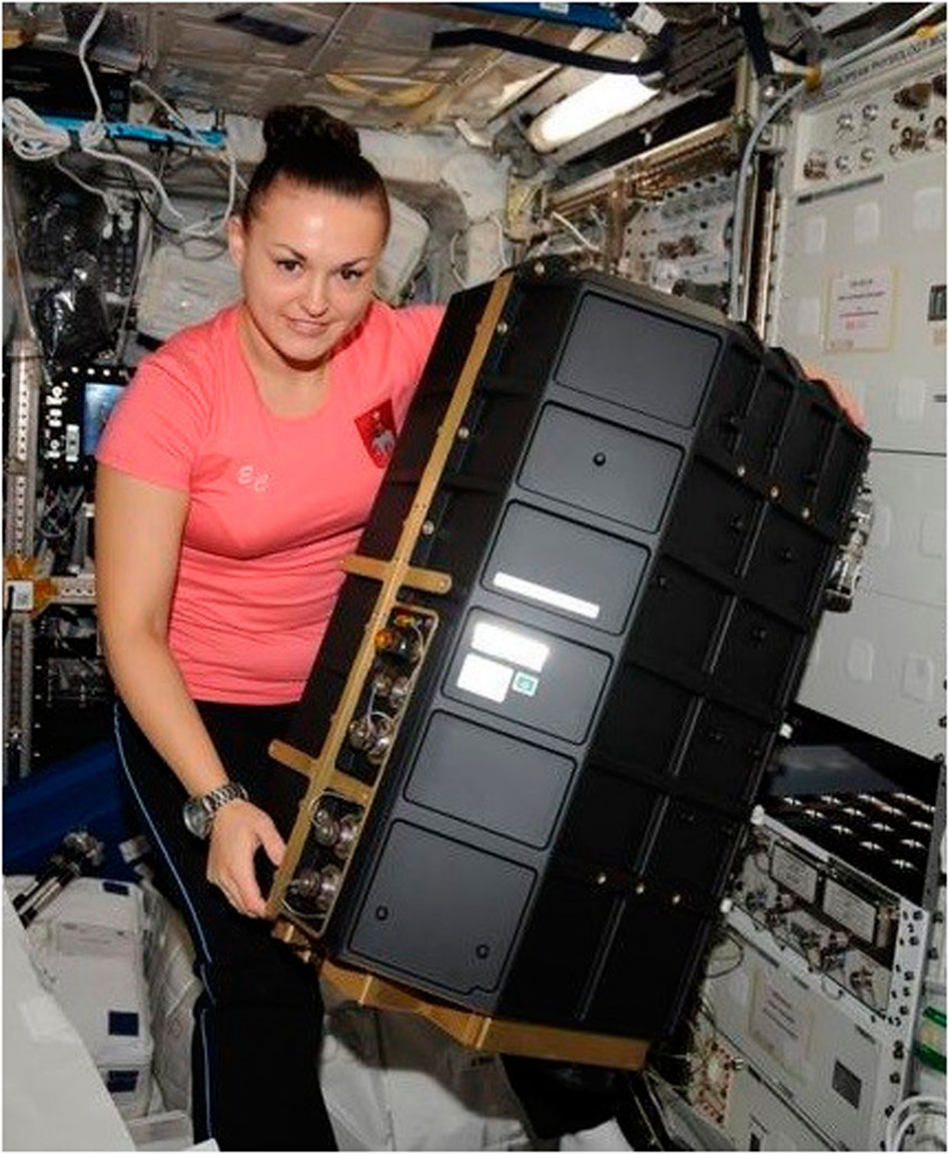
The PK-4 container onboard the ISS. PK-4 was installed in the Columbus module by Cosmonaut Elena Serova in November 2014 (Photo: DLR, CC BY-NC-ND 3.0).

The research on complex plasmas under microgravity conditions performed with the ISS-based laboratories PKE-Nefedov (2001–2005), PK-3 Plus (2006–2013) and PK-4 (since 2014, see Figs. 3 and 4)^31–33^ significantly contributed and still contributes to a better understanding of these systems. In total about 120 scientists from all over the world participated in these experiments leading to more than 130 peer reviewed publications. As examples, microgravity research on complex plasmas has opened up new topics like driven complex plasmas and phase separation in binary mixtures, phase transition from liquid to solid, electrorheological plasmas, etc.^31–43^. Besides these condensed matter topics, the research also provided great progress in the understanding of complex/dusty plasma specific topics, like waves, shock waves and Mach cones produced by projectiles, the charging/decharging of microparticles, the ion drag force, gelation and agglomeration of microparticles due to opposite charging, and many more^44–60^.

**Fig. 5:**
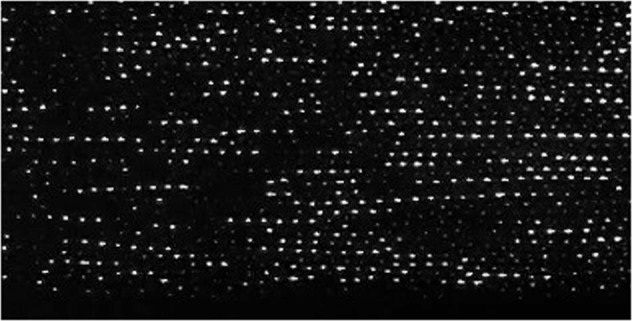
String formation. The micropartcles arrange in strings in the presence of an external electric field.

Presently the complex plasma facility PK-4 (“Plasmakristallexperiment #4”) is operated onboard the ISS. In contrast to its precursors, in which an rf discharge in a cubic plasma chamber was used to ignite the plasma, the plasma is produced by a dc discharge in an elongated glass tube (see Fig. 3). In addition, the PK-4 facility is equipped with various manipulation and diagnostic devices for performing many different experiments and providing a profound analysis. This configuration allows in particular the investigation of the liquid phase of the microparticle system, e.g., streaming of the microparticles, waves, electrorheology, turbulence, and viscosity. So far, 14 experiment campaigns have been conducted on the ISS leading already to numerous results e.g., refs. ^33,41–43,49–51,61^. A prominent example is the formation of an electrorheological plasma by applying an external electric field leading to the formation of microparticle strings (see Fig. 5)^41–43^. This experiment is possible only under microgravity where the microparticles are located in the center of the glass tube and not close to the bottom where the electric field of the plasma sheath prevents the string formation. Using ISS experiments the 3-dimensional structure of a string fluid and the propagation of dust waves in an electrorheological plasma were investigated^41^.

In the following, we will review future research opportunities with complex plasmas under microgravity. This review is based on a white paper prepared for the European Space Agency (ESA)^62^.

## Key knowledge gaps

New imaging technologies, current machine-learning based (image) analysis tools as well as newly developed techniques for generating near-equilibrium dust clouds allow to address new, relevant questions in the field of complex plasmas. The detailed investigations of these topics in 3-dimensional, homogeneous and isotropic complex plasmas are only possible under microgravity conditions.

Here, we list some hot topics as examples for key knowledge gaps in the field of complex plasmas as representative (model) system for other fields of physics:

1. Thermodynamics and Statistical physics: How does “breaking” Newton’s 3rd law affect the energy transport and temperature distribution in the system? In the presence of externally applied electric fields in complex plasmas the ions start to drift against the slow and heavy microparticles. This causes a wake potential with an interaction force that apparently leads to breaking Newton’s third law (actio = reactio) between the particles in this open system. This effect can lead to structural changes such as string formation in electrorheological plasmas^63^ (see Fig. 5). However, it is also of great interest to study the fundamental dynamical and thermodynamic properties of such a system, for which complex plasma provides an outstanding possibility. In laboratory experiments on quasi-2d systems some aspects like self-excited instabilities, non-equilibrium statistical mechanics or non-equilibrium thermodynamics have been observed and studied^64^. Under long-term and high-quality microgravity conditions, new aspects of statistical physics can be studied in 3D complex plasma systems, such as the measurement of the minimum value of the shear viscosity in the strongly coupled system, the validity of the work fluctuation theorem, or the equation of state that could be derived from measured 3D velocity distributions.
2. Phase transitions: How is the long-time dynamics in an undercooled liquid related to structural changes taking place close to the glass transition? The physics of undercooled liquids, especially near the glass transition, is one of the most controversially discussed topics in fluid physics^65–67^. There exist a number of mutually contradictory interpretations of different aspects of the complex behavior of undercooled liquids. For example, at least two different scenarios of dynamical heterogeneity are known, which lead to a stretched exponential relaxation at longer time scales. One theory links this to the spatial heterogeneity, another to fluctuations in the stochastic activation process. Another very important question is: what is the dependence of the structural glass transition on the spatial dimensionality (that is whether the system is 2D or 3D)? Especially what is the role of the geometrical frustration, which – as many believe – is essential for the glass transition^67^? Here, the ability of complex plasmas to resolve slow and fast dynamics on the individual particle level of thousands of particles in a 3D volume allows a unique view on these questions.
3. Hydrodynamics and Nonlinear dynamic: How do microscopic interactions lead to the development of large-scale nonlinear (turbulent) motion? Viscoelastic fluids, e.g., active fluids, polymer solutions with high viscosity, can exhibit turbulent behavior at very low Reynolds numbers. Hydrodynamic models describe the large-scale turbulent fluid motion, but cannot explain the underlying microscopic origin. Complex plasmas are a powerful tool for the investigation of fluids at “nanoscales”. Especially the investigation of the transition from the collective hydrodynamic behavior to the dynamics of individual particles is of special broader interest. The study of shear flow, vortex formation, viscoelastic properties^21,22,68^ of a wide range of coupling strength needs more fundamental investigation in the future and opens up one more interesting field of fundamental research in complex plasmas of basic interest (see Fig. 6). Additionally, a range of nonlinear wave phenomena can be studied in large 3D systems only under microgravity conditions, e.g., self-excited waves that appear when the dust density is increased above a critical value, the reflection behavior of dissipative solitary waves, or the propagation of shock waves.

**Fig. 6:**
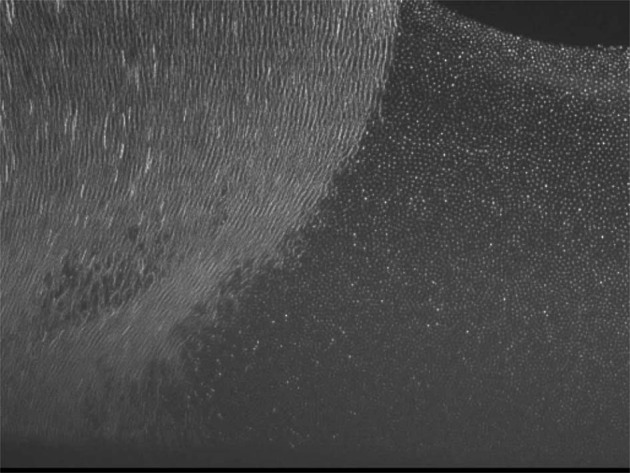
Turbulence in a complex plasma^83^. Turbulent microparticle flow has been observed in a complex plasma experiment.
4. Active and non-spherical particles in complex plasmas: How does the collective behavior of self-propelled particles connect to the energy input on the single-particle level? Anisotropic interaction can be forced due to the special shape of the particles. Rod-like particles, Janus particles with two different sides, platelets, cubes, pyramids, ellipsoids, etc. shall be mentioned here as examples^24,25^. In the context of active matter, complex plasmas may reveal the formation of various liquid and crystalline structures as well as dynamics characterized e.g., by non-Maxwellian velocity distributions^69,70^. In complex plasmas, where the interaction between the particles is long-range and the damping is very low (even the particle motion can be virtually undamped), active matter might exhibit new phenomena, which again can be studied on the most fundamental—the kinetic level. Active matter research is in need of experimental methods to provide realizations of large, dense, and tuneable 3D systems, which can be achieved with a complex plasma microgravity facility.
5. Natural dusty plasmas and planetary physics: Although mankind visited the Moon long time ago there are still a lot of questions about the dust of the lunar regolith: what is their charge in the local space plasma environment, are the local electric fields building up on the surface strong enough to transport, loft or even levitate dust, are the dust particles attracted by the surfaces of the space craft or space suits, etc.? Some of these questions need local in-situ measurements on the Moon but some aspects could be studied in a plasma chamber under lunar gravity parabolic flight conditions, like the charging of lunar dust simulants in a plasma, the lofting from a surface, their levitation and collective effects of larger dust clouds. This could give indications on the behavior of the dust on the Moon and strongly support the in-situ measurements. The investigations could be extended to dust on other planetary bodies or in planet forming regions. Further, plasma-based electrostatic methods to remove dust from surfaces, which are relevant for human exploration missions, show great promise and might require testing in microgravity environments.
6. Artificial intelligence and big data: Predictive science is in the forefront of aerodynamics, fluid dynamics, solid materials under extreme conditions, geology, biology and an increasing number of disciplines where complex phenomena are a common theme, in spite of the fact that the underlying processes and physics are simple and well understood. Data-driven and deep-learning methods are thriving in many of these areas. Such methods have already been applied to complex plasmas for structure recognition and stereoscopy^71–76^ and will be used much more in data analysis, classification and interpretation. The large video data sets of PK-4 and successors will require new efficient data processing, such as machine learning. They also will open completely new pathways, which are quite different from more established approaches such as human-intuition driven, first-physics-principle driven, and computationally driven methods. For example, machine learning models can be developed purely based on large experimental data sets^77–79^. Opportunities exist for predictive complex plasma science in the near future. This can be important also for automatic experiment control on the ISS to help obtain even better results. This basic knowledge would be easily transferable to other systems and to applications.

These questions have not been addressed in former ISS experiments either because it was not possible, e.g., because of missing active or non-spherical particles, or because other aspects have been studied. Whereas PKE-Nefedov and PK-3 Plus focussed on the study of the plasma crystal and related questions, PK-4 is used mainly to investigate the liquid phase of complex plasmas. The design of the plasma chambers in these experiments was adopted to these special applications. For the future a new experiment facility, called COMPACT (see below), will be designed in a way to tackle the important open problems listed above.

## Priorities for the space program (microgravity and/or Exploration relevance)

The priority of our research program in space is a better understanding of fundamental aspects of many-body physics as described above (topics 1–4) for which complex plasma is an ideal model system. In particular, the investigation of homogeneous 3D particle systems, which cannot be realized on Earth, will provide important new insights in fundamental questions concerning non-equilibrium thermodynamics, phase transitions, the origin of turbulence, active matter and others. Long-term investigations on platforms in Low-Earth-Orbit are the optimal means of choice for this research, whereas parabolic flights are useful for preparing the experiments.

The second priority is natural dusty plasmas (topic 5) as encountered in the lunar environment. Here parabolic flights under Moon gravity conditions should be considered, but also fundamental dusty plasma measurements on the Moon are necessary for detailed planning of dust mitigation.

## Benefit for Earth and industrial relevance

The knowledge gaps to be filled are current hot topics in the respective scientific fields, and great efforts are undertaken to answer them. The results are most relevant not only in science, but in industrial (e.g., turbulent flows in pipe constructions) and medical (e.g., self-propelled motion of living cells or driven protein filaments) applications as well, which would greatly profit from the expected knowledge gain. Lunar exploration missions are about to start and knowledge on the behavior and interactions of the charged dust component on the Moon’s surface is, therefore, important and timely.

## Recommendations in short, middle and long term

### Short term

The existing ISS facility PK-4 will be used for preliminary studies of the scientific program outlined above. The knowledge gained from these investigations concerning thermodynamics, phase transitions and hydrodynamics will be the starting point for further dedicated experiments in future microgravity facilities. PK-4 is the current laboratory installed in the Columbus module of the ISS and is planned to be operated at least until the beginning of 2023. It offers certain flexible possibilities for future research in complex plasma physics, but it is, due to its special design using a dc discharge plasma, not perfectly suited for the gross of topics mentioned above. Nevertheless, it offers a broad range of science topics to be studied as long as the main resources of microparticles and gas are available and the facility is operational.

### Middle/long term

The scientific program described above shall be considered by the development of a new facility “COMPACT” and its use on the ISS. A plasma laboratory for dedicated research topics is by definition always a multi-purpose and therefore a multi-user facility. The “COMPLEX PLASMA FACILITY” (COMPACT) could fulfill the requirements for the above-mentioned scientific topics, and even more. Its design builds upon the developments of the former Ekoplasma project^80,81^ and should be finalized in the near future. At the moment, a Phase A feasibility study is ongoing. In contrast to former microgravity experiments with complex plasmas the multi-purpose and multi-user facility COMPACT shall provide a much larger parameter space and much more flexible design allowing to address the key knowledge points mentioned in chapter 2 in great detail.

The heart of the facility COMPACT will be a cylindrical capacitive RF discharge plasma chamber, called Zyflex^81^. Capacitive RF discharges have already been used in PKE-Nefedov and PK-3 Plus. However, the Zyflex design will be quite different allowing to address new questions in complex plasma physics as the key knowledge gaps discussed above. First of all, the accessible parameter space will be enlarged greatly concerning electron temperature (0.1–6 eV compared to 2–3 eV in former experiments), plasma density (10^13^–10^15^ m^−3^ compared to 10^14^–10^16 ^m^−3^), and neutral gas pressure (0.1–100 Pa instead of 10–200 Pa). In addition, segmented electrodes will offer a much better local control of the plasma and the charged microparticles, and the distance between the electrodes can be changed between 25 and 75 mm. Improved optical diagnostics including a stereoscopic camera system will allow to measure the three-dimensional particle dynamics in real-time. Such data were never obtained in the former facilities and will yield important new insights relevant for all scientific topics to be addressed. Finally, new developments in the field of artificial intelligence for data compression, image evaluation and automatic experiment control shall be implemented allowing a much more flexible operation.

Another goal is the development of a dusty plasma program for the Moon. This combines investigations of lunar dust regolith interaction with plasma under lunar gravity parabolic flight conditions in short term and lunar dusty plasma in-situ measurements on the Moon in a separate ESA mission or bilateral or multi-lateral cooperation with USA and other partners in the middle to long term. The open fundamental questions and their space relevance and timelines are summarized in Table 1.

**Table 1:** Open fundamental scientific question for complex plasma research under microgravity.

Open fundamental scientific question	Future space experiments and suitable environment (LEO, Moon, Mars, BLEO)	Space relevance (importance of microgravity and/or relevance for space exploration)	Timeline (short, medium, long)
Thermodynamics	LEO (ISS)	Sedimentation - Study of large 3D systems only possible in micro-g	Medium to long (new hardware necessary)
Phase transitions	LEO (ISS)	Sedimentation - Study of large 3D systems only possible in micro-g	Medium to long (new hardware necessary)
Hydrodynamics	LEO (ISS)	Sedimentation - Study of large 3D systems only possible in micro-g	First experiments with PK-4: short; Medium to long (new hardware necessary)
Active particles in complex plasmas	LEO (ISS)	Sedimentation - Study of large 3D systems only possible in micro-g	Medium to long (new hardware necessary)
Natural dusty plasmas	Parabolic flights (luna-g), Moon	Especially dust lofting/levitation in a plasma needs to be investigated under lunar-g conditions on parabolic flights and directly on the Moon	Parabolic flights: short; Moon: medium, long

## Data Availability

The data that support the findings of this study are available from the corresponding author upon reasonable request.

## References

[1] Morfill GE, Ivlev AV (2009). Complex plasmas: an interdisciplinary research field. Rev. Mod. Phys..

[2] Fortov VE, Ivlev AV, Khrapak SA, Khrapak AG, Morfill GE (2005). Complex (dusty) plasmas: current status, open issues, perspectives. Phys. Rep..

[3] Piel A (2017). Plasma Crystals: experiments and simulations. Plasma Phys. Control. Fusion.

[4] Morfill GE, Ivlev AV, Thomas HM (2012). Complex (dusty) plasmas—kinetic studies of strong coupling phenomena. Phys. Plasmas.

[5] Melzer, A. *Physics of dusty plasmas: an introduction* (Springer Nature Switzerland, 2019).

[6] Nosenko V, Meyer J, Zhdanov SK, Thomas HM (2018). New radio-frequency setup for studying large 2D complex plasma crystals. AIP Adv..

[7] Homann A, Melzer A, Piel A (1999). Measuring the charge on single particles by laser-excited resonances in plasma crystals. Phys. Rev. E.

[8] Ratynskaia S (2004). Experimental determination of dust-particle charge in a discharge plasma at elevated pressures. Phys. Rev. Lett..

[9] Konopka U, Morfill GE, Ratke L (2000). Measurement of the interaction potential of microsphere in the sheath of a rf discharge. Phys. Rev. Lett..

[10] Zuzic M, Thomas HM, Morfill GE (1996). Wave propagation and damping in plasma crystals. J. Vac. Sci. Tech. A.

[11] Thomas E, Konopka U, Merlino RL, Rosenberg M (2016). Initial measurements of two- and three-dimensional ordering, waves, and plasma filamentation in the magnetized dusty plasma experiment. Phys. Plasmas.

[12] Schwabe M, Rubin-Zuzic M, Zhdanov S, Thomas HM, Morfill GE (2007). Highly resolved self-excited density waves in a complex plasma. Phys. Rev. Lett..

[13] Nosenko V, Goree J, Ma ZW, Piel A (2002). Observation of shear-wave Mach cones in a 2D dusty- plasma crystal. Phys. Rev. Lett..

[14] Samsonov D (1999). Mach cones in a Coulomb lattice and a dusty plasma. Phys. Rev. Lett..

[15] Melzer A, Homann A, Piel A (1996). Experimental investigation of the melting transition of the plasma crystal. Phys. Rev. E.

[16] Thomas HM, Morfill GE (1996). Melting dynamics of a plasma crystal. Nature.

[17] Knapek CA, Samsonov D, Zhdanov S, Konopka U, Morfill GE (2007). Recrystallization of a 2D plasma crystal. Phys. Rev. Lett..

[18] Rubin-Zuzic M (2006). Kinetic development of crystallization fronts in complex plasmas. Nat. Phys..

[19] Nosenko V, Zhdanov S, Morfill GE (2007). Supersonic dislocations observed in a plasma crystal. Phys. Rev. Lett..

[20] Nosenko V, Morfill GE, Rosakis P (2011). Direct experimental measurement of the speed-stress relation for dislocations in a plasma crystal. Phys. Rev. Lett..

[21] Nosenko V, Goree J (2004). Shear flows and shear viscosity in a two-dimensional Yukawa system (dusty plasma). Phys. Rev. Lett..

[22] Nosenko V, Ivlev AV, Morfill GE (2013). Anisotropic shear melting and recrystallization of a two-dimensional complex plasma. Phys. Rev. E.

[23] Nosenko V (2008). Heat transport in a two-dimensional complex (dusty) plasma at melting conditions. Phys. Rev. Lett..

[24] Annaratone BM (2001). Levitation of cylindrical particles in the sheath of an rf plasma. Phys. Rev. E.

[25] Nosenko V, Luoni F, Kaouk A, Rubin-Zuzic M, Thomas HM (2020). Active Janus particles in a complex plasma. Phys. Rev. Res..

[26] Ludwig P (2018). Non-Maxwellian and magnetic field effects in complex plasma wakes. Eur. Phys. J. D..

[27] Piel A (2018). Microphysics of liquid complex plasmas in equilibrium and non-equilibrium systems. Eur. Phys. J. D..

[28] Piel A, Greiner F, Jung H, Miloch WJ (2018). Molecular dynamics simulations of wake structures behind a microparticle in a magnetized ion flow. I. Collisionless limit with cold ion beam. Phys. Plasmas.

[29] Piel A, Jung H, Greiner F (2018). Molecular dynamics simulations of wake structures behind a microparticle in a magnetized ion flow. II. Effects of velocity spread and ion collisions. Phys. Plasmas.

[30] Block D, Melzer A (2019). Dusty (complex) plasmas—routes towards magnetized and polydisperse systems. J. Phys. B: . Mol. Opt. Phys..

[31] Nefedov AP (2003). PKE-Nefedov: plasma crystal experiments on the International Space Station. N. J. Phys..

[32] Thomas HM (2008). Complex plasma laboratory PK-3 Plus on the International Space Station. N. J. Phys..

[33] Pustylnik MY (2016). Plasmakristall-4: New complex (dusty) plasma laboratory on board the International Space Station. Rev. Sci. Instrum..

[34] Sütterlin KR (2009). Dynamics of lane formation in driven binary complex plasmas. Phys. Rev. Lett..

[35] Khrapak SA (2011). Freezing and melting of 3D complex plasma structures under microgravity conditions driven by neutral gas pressure manipulation. Phys. Rev. Lett..

[36] Naumkin VN (2018). Crystal–liquid phase transitions in three-dimensional complex plasma under microgravity conditions. J. Phys.: Conf. Ser..

[37] Ivlev AV, Zhdanov SK, Thomas HM, Morfill GE (2009). Fluid phase separation in binary complex plasmas. EPL.

[38] Killer C (2016). Phase separation of binary charged particle systems with small size disparities using a dusty plasma. Phys. Rev. Lett..

[39] Schütt S, Himpel M, Melzer A (2020). Experimental investigation of phase separation in binary dusty plasmas under microgravity. Phys. Rev. E.

[40] Ivlev AV (2008). First observation of electrorheological plasmas. Phys. Rev. Lett..

[41] Pustylnik MY (2020). Three-dimensional structure of a string-fluid complex plasma. Phys. Rev. Res..

[42] Nosenko V (2020). Shear flow in a three-dimensional complex plasma in microgravity conditions. Phys. Rev. Res..

[43] Schwabe M (2020). Slowing of acoustic waves in electrorheological and string-fluid complex plasmas. N. J. Phys..

[44] Khrapak S (2003). Compressional waves in complex (dusty) plasmas under microgravity conditions. Phys. Plasmas.

[45] Samsonov D (2003). Kinetic measurements of shock wave propagation in a three-dimensional complex (dusty) plasma. Phys. Rev. E.

[46] Yaroshenko VV (2004). Electrostatic modes in collisional complex plasmas under microgravity conditions. Phys. Rev. E.

[47] Schwabe M (2008). Nonlinear waves externally excited in a complex plasma under microgravity conditions. N. J. Phys..

[48] Jiang K (2009). Mach cones in a three-dimensional complex plasma. EPL.

[49] Jaiswal S (2018). Dust density waves in a dc flowing complex plasma with discharge polarity reversal. Phys. Plasmas.

[50] Yaroshenko VV (2019). Excitation of low-frequency dust density waves in flowing complex plasmas. Phys. Plasmas.

[51] Antonova T (2019). Particle charge in PK-4 dc discharge from ground-based and microgravity experiments. Phys. Plasmas.

[52] Ivlev AV (2003). Decharging of complex plasmas: First kinetic observations. Phys. Rev. Lett..

[53] Khrapak SA, Ivlev AV, Morfill, Thomas HM (2002). Ion drag force in complex plasmas. Phys. Rev. E.

[54] Ivlev AV, Morfill GE, Konopka U (2002). Coagulation of charged microparticles in neutral gas and charge-induced gel transitions. Phys. Rev. Lett..

[55] Konopka U (2005). Charge-induced gelation of microparticles. N. J. Phys..

[56] Bin L (2021). Nonlinear wave synchronization in a dusty plasma under microgravity on the International Space Station (ISS). IEEE Trans. Plasma Sci..

[57] Himpel M (2014). Stereoscopy of dust density waves under microgravity: Velocity distributions and phase-resolved single-particle analysis. Phys. Plasmas.

[58] Himpel M, Killer C, Buttenschön B, Melzer A (2012). Three-dimensional single particle tracking in dense dust clouds by stereoscopy of fluorescent particles. Phys. Plasmas.

[59] Buttenschön B, Himpel M, Melzer A (2011). Spatially resolved three-dimensional particle dynamics in the void of dusty plasmas under microgravity using stereoscopy. N. J. Phys..

[60] Wolter M, Melzer A, Arp O, Klindworth M, Piel A (2007). Force measurements in dusty plasmas under microgravity by means of laser manipulation. Phys. Plasmas.

[61] Kretschmer M, Antonova T, Zhdanov S, Thoma MH (2016). Wave phenomena in a stratified complex plasma. IEEE Trans. Plasma Sci..

[62] Vailati, A. et al. ROADMAP: Soft Matter and Biophysics, https://esamultimedia.esa.int/docs/HRE/04_Physical_Sciences_Soft-Matter-Biophysics.pdf (2021).

[63] Ivlev AV (2015). Statistical mechanics where newton’s third law is broken. Phys. Rev. X.

[64] Wong C, Goree J, Haralson Z, Bin L (2018). Strongly coupled plasmas obey the fluctuation theorem for entropy production. Nat. Phys..

[65] Jäckle J (1986). Models of the glass transition. Rep. Prog. Phys..

[66] March, N. H. & Tosi, M. P. *Introduction to Liquid State Physics* (World Scientific, 2002).

[67] Shintani H, Tanaka H (2006). Frustration on the way to crystallization in glass. Nat. Phys..

[68] Feng Y, Goree J, Bin L (2012). Observation of temperature peaks due to strong viscous heating in a dusty plasma flow. Phys. Rev. Lett..

[69] Gupta A, Ganesh R (2020). The emergence of inertial waves from coherent vortex source in strongly coupled dusty plasma. Phys. Plasmas.

[70] Mulsow M, Himpel M, Melzer A (2017). Analysis of 3D vortex motion in a dusty plasma. Phys. Plasmas.

[71] Dietz C, Kretz T, Thoma MH (2017). Machine-learning approach for local classification of crystalline structures in multiphase systems. Phys. Rev. E.

[72] Williams JD (2011). Application of tomographic particle image velocimetry to studies of transport in complex (dusty) plasma. Phys. Plasmas.

[73] Himpel M, Melzer A (2019). Three-dimensional reconstruction of individual particles in dense dust clouds: benchmarking camera orientations and reconstruction algorithms. J. Imaging.

[74] Himpel M, Melzer A (2021). Fast 3D particle reconstruction using a convolutional neural network: application to dusty plasmas. Mach. Learn. Sci. Technol..

[75] Huang H, Schwabe M, Du C-R (2019). Identification of the interface in a binary complex plasma using machine learning. J. Imaging.

[76] Dietz C, Budak J, Kamprich T, Kretschmer M, Thoma MH (2021). Phase transition in electrorheological plasmas. Contrib. Plasma Phys..

[77] Rouet-Leduc B (2017). Machine learning predicts laboratory earthquakes. Geophys. Res. Lett..

[78] Radovic A (2018). Machine learning at the energy and intensity frontiers of particle physics. Nature.

[79] Biswas R (2013). Application of machine learning algorithms to the study of noise artifacts in gravitational-wave data. Phys. Rev. D..

[80] Knapek CA (2018). Ekoplasma—Experiments with grid electrodes in microgravity. AIP Conf. Proc..

[81] Knapek CA (2021). “Zyflex”: next generation plasma chamber for complex plasma research in space. Rev. Sci. Instrum..

[82] Thomas HM (2005). PKE-Nefedov—complex plasma research on the international space station. Microgravity Sci. Technol..

[83] Morfill GE (2004). “Liquid plasmas” at the kinetic level. Phys. Rev. Lett..

